# Body mass index and mortality in patients with schizophrenia spectrum disorders: a cohort study in a South London catchment area

**DOI:** 10.1136/gpsych-2022-100819

**Published:** 2022-11-04

**Authors:** Jianhua Chen, Gayan Perera, Hitesh Shetty, Matthew Broadbent, Yifeng Xu, Robert Stewart

**Affiliations:** 1 Psychological Medicine, Institute of Psychiatry, Psychology & Neuroscience, King's College London, London, UK; 2 Shanghai Clinical Research Center for Mental Health, Shanghai Key Laboratory of Psychotic Disorders, Shanghai Mental Health Center, Shanghai Jiao Tong University School of Medicine, Shanghai, China; 3 Biomedical Research Centre, South London and Maudsley NHS Foundation Trust, London, UK

**Keywords:** schizophrenia, mental health services, life style

## Abstract

**Background:**

People with schizophrenia have a high premature mortality risk. Obesity is a key potential underlying risk factor that is relatively unevaluated to date.

**Aims:**

In this study, we investigated the associations of routinely recorded body size with all-cause mortality and deaths from common causes in a large cohort of people with schizophrenia spectrum disorders.

**Methods:**

We assembled a retrospective observational cohort using data from a large mental health service in South London. We followed all patients over the age of 18 years with a clinical diagnosis of schizophrenia spectrum disorders from the date of their first recorded body mass index (BMI) between 1 January 2007 and 31 March 2018.

**Results:**

Of 11 900 patients with a BMI recording, 1566 died. The Cox proportional hazards regression models, after adjusting for sociodemographic, socioeconomic variables and comorbidities, indicated that all-cause mortality was only associated with underweight status compared with healthy weight status (hazard ratio (HR): 1.33, 95% confidence interval (CI): 1.01 to 1.76). Obesity (HR: 1.24, 95% CI: 1.01 to 1.52) and morbid obesity (HR: 1.54, 95% CI: 1.03 to 2.42) were associated with all-cause mortality in the 18–45 years age range, and obesity was associated with lower risk (HR: 0.66, 95% CI: 0.50 to 0.87) in those aged 65+ years. Cancer mortality was raised in underweight individuals (HR: 1.93, 95% CI: 1.03 to 4.10) and respiratory disease mortality raised in those with morbid obesity (HR: 2.17, 95% CI: 1.02 to 5.22).

**Conclusions:**

Overall, being underweight was associated with higher mortality in this disorder group; however, this was potentially accounted for by frailty in older age groups, and obesity was a risk factor for premature mortality in younger ages. The impact of obesity on life expectancy for people with schizophrenia spectrum disorders is clear from our findings. A deeper biological understanding of the relationship between these diseases and schizophrenia will help improve clinical practice.

**WHAT IS ALREADY KNOWN ON THIS TOPIC**

- Higher body mass index in overweight or obese ranges potentially accounts for at least some of the premature mortality experienced by individuals with schizophrenia spectrum disorders.

**WHAT THIS STUDY ADDS**

- Obesity was associated with higher mortality risk in patients aged 18–45 years; however, underweight status was the predominant risk factor in the sample overall, particularly noticeable in patients over 65 years old.

**HOW THIS STUDY MIGHT AFFECT RESEARCH, PRACTICE OR POLICY**

- Although continued attention is needed to reduce obesity in people with schizophrenia spectrum disorders, more attention needs to be paid to underweight status as a risk factor for mortality, particularly in older age groups. For example, clarifying whether this association simply reflects underlying comorbidity or whether there are causal pathways relating to the mental disorder itself (eg, self-neglect) might need further investigation.

## Introduction

Schizophrenia is a severe and often longstanding mental disorder that affects about 1% of the global population.^1^ People with schizophrenia often face health, economic and social challenges, which may have negative impacts on prognosis and survival.^2^ Excess mortality is experienced by patients with all severe mental disorders across all age groups, including premature mortality in people with schizophrenia,^3^ and a decrease in life expectancy by approximately 15–25 years.^4^ Approximately 60% of the excess deaths are related to comorbid physical illnesses, especially chronic diseases such as type 2 diabetes mellitus, cardiovascular disease, respiratory diseases, stroke and some cancers.^5^ Cardiovascular disease is a leading cause of death and is twofold to threefold higher in patients with schizophrenia compared with the general population.^6^ The increased risk of cardiovascular disease involves multiple factors, including higher smoking rates, low income, unhealthy diet and decreased physical activity.^7^ Antipsychotic medications also have the potential to induce metabolic syndrome, which is a key risk factor for cardiovascular disease and is present at a fourfold higher prevalence in patients with schizophrenia than the general population.^8^

Obesity is a common feature of metabolic syndrome, and individuals with schizophrenia have a threefold higher risk of obesity.^9^ It is estimated that 40%–60% of people with psychotic spectrum disorders are overweight or obese, which is significantly higher than the overall population.^10^ Patients with obesity had higher glucose, triglycerides, cholesterol and low-density lipoprotein levels, but lower high-density lipoprotein levels, all of which indicate a greater risk of cardiovascular disease. Genetics, pathophysiology, lifestyle factors and medications may contribute to the higher obesity rates associated with schizophrenia. For instance, in patients with schizophrenia, olanzapine and clozapine are the most commonly used atypical antipsychotics that may increase the intake of food or lead to oxidative damage, causing obesity.^11^ Obesity impacts physical health and mortality, and it may also affect mental health and brain structure.^12^ There is some evidence that obesity may worsen schizophrenia symptoms by disrupting white matter and reducing brain structural connectivity.^11^

However, underweight status is also associated with health risks, and a low body mass index (BMI) can also reflect self-neglect or physical frailty. To our knowledge, few studies have investigated body size as a specific risk factor for mortality in people with schizophrenia spectrum disorders despite the recognised importance of obesity as a factor potentially underlying health inequality. In this study, we sought to investigate associations of routinely recorded body size with all-cause mortality, and with deaths from common causes, in a large cohort of people with schizophrenia spectrum disorders.

## Methods

### Setting and study population

We assembled a retrospective observational cohort using data from the South London and Maudsley National Health Service Foundation Trust (SLaM) Clinical Record Interactive Search (CRIS) platform.^13^ SLaM provides specialist mental health services to approximately 1.3 million residents of a defined geographical catchment area in southeast London, and is one of the largest mental healthcare providers in Europe.^13^ From 2006 onwards, electronic health records have been used across all SLaM services, and in 2008, the CRIS application was developed to enable researcher access to de-identified data derived from the health records within a robust governance framework.^14^ Over 500 000 cases are currently archived on CRIS and the database has been approved for secondary analysis.^13^ CRIS includes data extracts from unstructured fields (eg, clinical notes, clinic letters) using the Generalised Architecture for Text Engineering (GATE) software. The GATE software supports a wide range of natural language processing (NLP) algorithms to identify entities from text related to diagnosis, treatment, symptoms, lifestyle factors and other relevant clinical information.^13^ The SLaM case register derived from CRIS has been previously described in detail, and has been used to address a range of research hypotheses in mental health.^13^

For this study, we assembled a sample of all patients over the age of 18 years who had received a clinical diagnosis of schizophrenia spectrum disorder at any time between 1 January 2007 and 31 March 2018. The clinical diagnoses were ascertained from World Health Organization (WHO) International Classification of Diseases, 10th Revision (ICD-10) codes in a compulsory structured field within the record. All patients with an ICD-10 F2x diagnostic code (schizophrenia, schizotypal and delusional disorders) were included. The sample was further restricted to those with at least one recorded BMI score within a feasible range (10–50); 11 281 patients were excluded from the study as they did not have at least one BMI value. We ascertained the date of first recorded BMI (defined as the index date; see below for ascertainment) and BMI value after diagnosis of schizophrenia spectrum disorder; 11 900 patients with at least one BMI value were eventually included in the study.

### Exposure and outcome

Body size was obtained through a bespoke NLP algorithm with a precision of 89% (positive predictive value) that was applied to the entire CRIS Database.^15^ Extracted scores were obtained from the first available records after 2007 and the following standard categories were applied: (1) healthy weight (BMI: 18.5–24); (2) underweight (BMI: <18.5); (3) overweight (BMI: 25–29); (4) obese (BMI: 30–39); (5) morbidly obese (BMI: 40+).

The primary outcome was the mortality ascertained over the 11-year observation window (2007–2018) from a linkage to national death registrations held by the Office for National Statistics, available up to 27 April 2018 at the time of analysis. In addition, the cause of death according to ICD-10 code was ascertained based on the listed underlying primary cause and categorised as follows: cancers and neoplasms (Chapter C), diseases of the circulatory system (Chapter I), diseases of the respiratory system (Chapter J), diseases of the digestive system (Chapter K), external causes (Chapters S, T, V, W, X, Y, Z) and all other causes.

### Covariates

We obtained the following information applied to the index date: age, gender, ethnicity (white vs non-white), living alone, marital status (cohabiting vs non-cohabiting) and smoking status (current smoker, ex-smoker, non-smoker). We estimated neighbourhood socioeconomic status using the Index of Multiple Deprivation (IMD) (2015) projections from the UK Census at the level of the Lower Super Output Area (a standard residential unit with an average population of 1500) at the index date.^16^ Employment status closest to the index date was categorised as disabled, employed or student, retired, unemployed or other.

The Health of the Nation Outcome Scale (HoNOS) is a 12-subscale assessment of health and function routinely administered in UK mental health services.^17^ The following 11-subscale scores were extracted closest to the index date: agitation, self-injury, drinking problems, cognition, physical illness, hallucinations, depression, relationships, activities of daily living, living conditions and occupation.^18^ Subscale scores range from 0 (no problem) to 4 (severe problem); scores of 2 and over were classified as a problem for each subscale and defined as binary variables for this analysis.

Data on recorded medication use within 12 months before and after the index date were extracted from the structured medication fields in the record, supplemented by NLP algorithms applied to text domains.^13^ Medications extracted comprised the following groups: antipsychotics, antidepressants, anxiolytics/hypnotics, mood stabilisers, antihypertensives, lipid-lowering medication and antidiabetic medication. Furthermore, we obtained information on hospital admissions 12 months before and after the index date from linked national hospitalisation data; these were defined using ICD-10 discharge diagnosis data to ascertain hospitalised diabetes (E08–14), hypertension (I10–15) and hyperlipidaemia (E78).

### Statistical analysis

The study population which was not included in the final study cohort (n=11 281) and the final study cohort (n=11 900) were compared by sociodemographics, smoking status, medications received and hospitalisation. To compare the two groups, a χ^2^ test, Student’s t-test and difference in proportion tests were used (online supplemental table).

10.1136/gpsych-2022-100819.supp1 Supplementary data

Hazard ratios (HRs) were calculated using Cox proportional hazards models for the risk of death for all age groups, next stratified by age group, and finally by the primary underlying cause of death category in the death record. The censoring point for Cox models was 27 April 2018.

Age was a covariate in all regression analyses. The final fully adjusted model was adjusted for age, gender, marital status, ethnicity, employment status, IMD score, smoking status, individual HoNOS problems, medication groups, and hospitalised diabetes, hypertension and hyperlipidaemia.

Furthermore, Cox proportional HRs were calculated for all-cause mortality by baseline recorded BMI stratified by median follow-up time which was 6.385 years.

All analyses were performed using Stata V.13 (StataCorp, College Station, Texas, USA) and the statistical significance threshold was set as 0.05.

## Results

**Figure 1:**
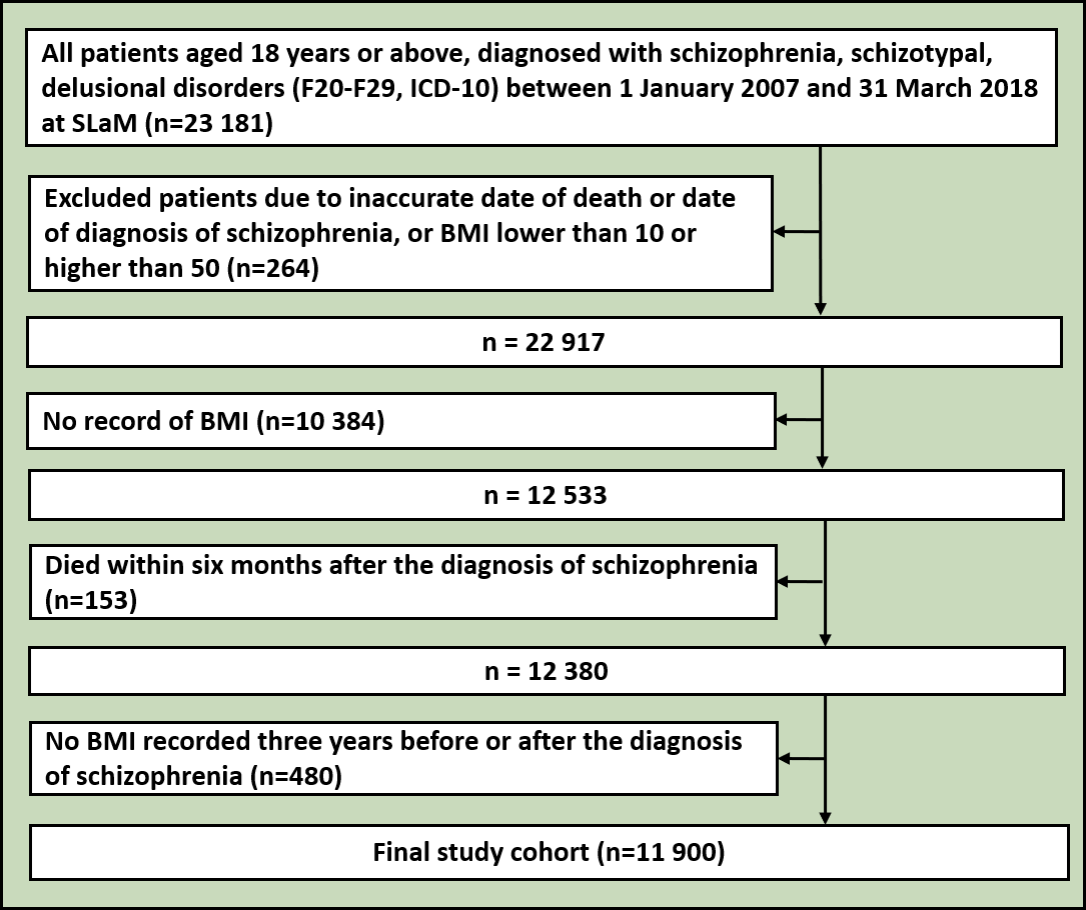
Flowchart of this study. BMI, body mass index; ICD-10, International Classification of Diseases, 10th Revision; NHS, National Health Service; SLaM, South London and Maudsley NHS Foundation Trust.

From 1 January 2007 to 31 March 2018, we ascertained 23 181 cases of schizophrenia spectrum disorders in CRIS, 3660 of whom had died during the follow-up period. Of these, 11 281 patients were excluded because of insufficient BMI data, resulting in an analysed sample of 11 900 patients of whom 1566 died (figure 1). A comparison of the groups that were and were not included in the final study cohort showed that the non-included group had higher mortality, were older, more often white and less likely to have problems with most HoNOS. Additionally, they were seldom on psychotropic treatment (online supplemental table). Of the analysed cohort (table 1), those in the overweight/obese groups were older, from non-white ethnic groups, unemployed and in higher deprivation neighbourhoods; those in the healthy and overweight groups were more likely to be male. Current smoking status was more likely in the healthy weight and underweight groups. In terms of HoNOS problems, the most marked differences were seen in alcohol/substance use problems (highest in those with healthy weight or underweight), physical health problems (highest in the obese and morbidly obese groups), hallucinations/delusions (highest in the healthy weight and underweight groups), activities of daily living problems (highest in the underweight and morbidly obese groups), living condition and occupation problems (highest in the underweight group). Hypertension, hyperlipidaemia and diabetes hospitalisation, and medications for these conditions were more common in the more obese groups. The highest proportions of deaths were observed in the underweight group and the lowest proportions in the healthy weight group.

**Table 1:** Characteristics of the study cohort

Characteristics	Healthy weight (n=4659)	Underweight (n=555)	Overweight (n=3403)	Obese (n=2782)	Morbidly obese (n=501)
Age at baseline (%)					
18–24	1017 (21.8)	116 (20.9)	445 (13.1)	253 (9.1)	36 (7.2)
25–34	1216 (26.1)	142 (25.6)	825 (24.2)	620 (22.3)	106 (21.2)
35–44	990 (21.2)	117 (21.1)	871 (25.6)	770 (27.7)	153 (30.5)
45–54	673 (14.4)	67 (12.1)	586 (17.2)	599 (21.5)	137 (27.3)
55–64	335 (7.2)	37 (6.7)	324 (9.5)	285 (10.2)	43 (8.6)
65–74	254 (5.5)	36 (6.5)	216 (6.3)	164 (5.9)	22 (4.4)
75–84	135 (2.9)	30 (5.4)	112 (3.3)	80 (2.9)	2 (0.4)
85 and above	39 (0.8)	10 (1.8)	24 (0.7)	11 (0.4)	2 (0.4)
Mean (SD) age at baseline	39.2 (16.1)	40.8 (18.2)	42.3 (15.8)	43.4 (14.6)	42.4 (12.4)
Gender (%)					
Female	1651 (35.4)	295 (53.2)	1249 (36.7)	1345 (48.3)	308 (61.5)
Male	3008 (64.6)	260 (46.8)	2154 (63.3)	1437 (51.7)	193 (38.5)
Cohabiting status (%)					
Cohabiting	409 (8.8)	50 (9.0)	433 (12.7)	340 (12.2)	61 (12.2)
Non-cohabiting	4250 (91.2)	505 (91.0)	2970 (87.3)	2442 (87.8)	440 (87.8)
Living alone (%)	1075 (23.1)	126 (22.7)	801 (23.5)	656 (23.6)	103 (20.6)
Ethnicity (%)					
Non-white	2565 (55.1)	293 (52.8)	2020 (59.4)	1647 (59.2)	295 (58.9)
White	2048 (44.0)	258 (46.5)	1360 (40.0)	1117 (40.2)	203 (40.5)
Missing data	46 (1.0)	4 (0.7)	23 (0.7)	18 (0.6)	3 (0.6)
Employment status (%)					
Employed or student	219 (4.7)	29 (5.2)	145 (4.3)	121 (4.3)	23 (4.6)
Unemployed	1225 (26.3)	131 (23.6)	1077 (31.6)	1067 (38.4)	212 (42.3)
Retired	117 (2.5)	22 (4.0)	122 (3.6)	86 (3.1)	9 (1.8)
Disabled	5 (0.1)	1 (0.2)	3 (0.1)	16 (0.6)	1 (0.2)
Other	1202 (25.8)	130 (23.4)	951 (27.9)	837 (30.1)	154 (30.7)
Missing data	1891 (40.6)	242 (43.6)	1105 (32.5)	655 (23.5)	102 (20.4)
Mean (SD) Index of Multiple Deprivation	30.9 (9.8)	30.5 (9.5)	31.4 (9.5)	31.2 (9.6)	31.8 (9.6)
Smoking status (%)					
Current smoker	3205 (68.8)	380 (68.5)	2236 (65.7)	1739 (62.5)	293 (58.5)
Ex-smoker	168 (3.6)	20 (3.6)	119 (3.5)	117 (4.2)	24 (4.8)
Non-smoker	1220 (26.2)	147 (26.5)	1001 (29.4)	887 (31.9)	177 (35.3)
Missing data	66 (1.4)	8 (1.4)	47 (1.4)	39 (1.4)	7 (1.4)
HoNOS problem (%)					
Agitation	1281 (27.5)	148 (26.7)	827 (24.3)	656 (23.6)	124 (24.8)
Self-injury	364 (7.8)	40 (7.2)	197 (5.8)	143 (5.1)	35 (7.0)
Drinking	1093 (23.5)	111 (20.0)	577 (17.0)	393 (14.1)	43 (8.6)
Cognition	936 (20.1)	112 (20.2)	667 (19.6)	517 (18.6)	101 (20.2)
Physical illness	753 (16.2)	117 (21.1)	623 (18.3)	630 (22.6)	168 (33.5)
Hallucinations	2531 (54.3)	305 (55.0)	1783 (52.4)	1416 (50.9)	239 (47.7)
Depression	1211 (26.0)	156 (28.1)	799 (23.5)	614 (22.1)	132 (26.3)
Relationships	1732 (37.2)	205 (36.9)	1214 (35.7)	963 (34.6)	171 (34.1)
Daily living	1360 (29.2)	197 (35.5)	960 (28.2)	832 (29.9)	183 (36.5)
Living conditions	1098 (23.6)	157 (28.3)	755 (22.2)	536 (19.3)	94 (18.8)
Occupation	1504 (32.3)	194 (34.9)	1070 (31.4)	805 (28.9)	138 (27.5)
Psychiatric medication (%)					
Antipsychotic	4223 (90.6)	494 (89.0)	3131 (92.0)	2548 (91.6)	457 (91.2)
Antidepressant	1776 (38.1)	235 (42.3)	1383 (40.6)	1173 (42.2)	216 (43.1)
Anxiolytic/hypnotic	2968 (63.7)	330 (59.5)	2009 (59.0)	1598 (57.4)	284 (56.7)
Mood stabiliser	728 (15.6)	45 (8.1)	672 (19.7)	668 (24.0)	130 (25.9)
Medication for CVD (%)					
Antihypertensive	43 (0.9)	7 (1.3)	71 (2.1)	62 (2.2)	19 (3.8)
Lipid lowering	285 (6.1)	34 (6.1)	364 (10.7)	409 (14.7)	87 (17.4)
Antidiabetic	114 (2.4)	20 (3.6)	157 (4.6)	183 (6.6)	44 (8.8)
Hospitalised circulatory diseases (%)					
Diabetes	110 (2.4)	13 (2.3)	148 (4.3)	167 (6.0)	40 (8.0)
Hypertension	160 (3.4)	21 (3.8)	183 (5.4)	197 (7.1)	57 (11.4)
Hyperlipidaemia	72 (1.5)	5 (0.9)	72 (2.1)	78 (2.8)	19 (3.8)
Deceased (%)	580 (12.4)	98 (17.7)	423 (12.4)	394 (14.2)	71 (14.2)
Mean (SD) years of follow-up	4.1 (2.9)	3.7 (2.6)	4.2 (3.0)	4.8 (3.2)	4.2 (2.7)
Death by underlying cause (%)					
Circulatory	100 (2.1)	15 (2.7)	74 (2.2)	93 (3.3)	9 (1.8)
Cancer	81 (1.7)	6 (1.1)	69 (2.0)	37 (1.3)	7 (1.4)
Respiratory	60 (1.3)	19 (3.4)	43 (1.3)	37 (1.3)	7 (1.4)
Digestive	32 (0.7)	6 (1.1)	17 (0.5)	12 (0.4)	4 (0.8)
External causes	65 (1.4)	13 (2.3)	38 (1.1)	35 (1.3)	7 (1.4)
Type of external cause*(%)					
Accidents	27 (0.6)	7 (1.3)	17 (0.5)	14 (0.5)	5 (1.0)
Suicide or self-harm	25 (0.5)	2 (0.4)	15 (0.4)	12 (0.4)	2 (0.4)
Unknown intent	13 (0.3)	4 (0.7)	6 (0.2)	9 (0.3)	0 (0.0)
Other causes	242 (5.2)	39 (7.0)	182 (5.3)	180 (6.5)	37 (7.4)

*External causes were divided into accidents, suicide or self-harm, unknown intent or other causes according to ICD-10 description.

CVD, cardiovascular disease; HoNOS, Health of the Nation Outcome Scale; ICD-10, International Classification of Diseases, 10th Revision; SD, standard deviation.

HRs for all-cause mortality are displayed in table 2. In the fully adjusted model, compared with the healthy weight group, mortality was significantly raised in the underweight group (HR: 1.33, 95% CI: 1.01 to 1.76) and lowered in the overweight group (HR: 0.87, 95% CI: 0.75 to 0.99), with close to null associations in the obese and morbidly obese groups. When stratified by age, both obesity (HR: 1.24, 95% CI: 1.01 to 1.52) and morbid obesity (HR: 1.54, 95% CI: 1.03 to 2.42) were significantly associated with mortality among the 18–45 years age group, whereas associations with underweight status were close to the null. In contrast, in the group aged 65+ years, underweight status was strongly associated with higher mortality (HR: 1.77, 95% CI: 1.17 to 2.67) and obesity with lower mortality (HR: 0.66, 95%CI: 0.50 to 0.87).

**Table 2:** Cox proportional hazards model used for all-cause mortality by baseline recorded BMI

Age group	Adjusted for age HR (95% CI), P value	Fully adjusted model HR (95% CI), P value
All ages		
Healthy weight	Reference	Reference
Underweight	1.32 (1.06 to 1.63), 0.012	1.33 (1.01 to 1.76), 0.042
Overweight	0.83 (0.74 to 0.95), <0.001	0.87 (0.75 to 0.99), 0.046
Obese	0.91 (0.80 to 1.04), 0.152	0.90 (0.77 to 1.05), 0.175
Morbidly obese	1.12 (0.88 to 1.44), 0.354	1.01 (0.77 to 1.35), 0.926
18–45		
Healthy weight	Reference	Reference
Underweight	1.11 (0.69 to 1.78), 0.676	0.78 (0.40 to 1.49), 0.447
Overweight	0.94 (0.73 to 1.21), 0.637	0.92 (0.69 to 1.23), 0.576
Obese	1.28 (1.03 to 1.63), 0.036	1.24 (1.01 to 1.52), 0.047
Morbidly obese	1.75 (1.18 to 2.61), 0.012	1.54 (1.03 to 2.42), 0.036
45–64		
Healthy weight	Reference	Reference
Underweight	1.34 (0.89 to 2.01), 0.163	1.41 (0.85 to 2.34), 0.182
Overweight	0.74 (0.60 to 0.92), 0.008	0.79 (0.61 to 1.01), 0.056
Obese	0.88 (0.72 to 1.09), 0.247	0.90 (0.71 to 1.15), 0.416
Morbidly obese	0.86 (0.58 to 1.27), 0.462	0.66 (0.41 to 1.06), 0.087
65+		
Healthy weight	Reference	Reference
Underweight	1.44 (1.07 to 1.95), 0.017	1.77 (1.17 to 2.67), 0.009
Overweight	0.87 (0.71 to 1.05), 0.152	0.83 (0.65 to 1.06), 0.129
Obese	0.73 (0.59 to 0.91), 0.012	0.66 (0.50 to 0.87), <0.001
Morbidly obese	1.09 (0.62 to 1.90), 0.766	1.44 (0.75 to 2.77), 0.284

Fully adjusted model adjusted for age, gender, marital status, ethnicity, employment status, IMD score, smoking status, HoNOS problems, received antipsychotic, antidepressant, manic, antihypertensives, lipid-lowering and antidiabetic medications, and comorbidities of diabetes, hypertension and hyperlipidaemia.

BMI, body mass index; CI, confidence interval; HoNOS, Health of the Nation Outcome Scale; HR, hazard ratio; IMD, Index of Multiple Deprivation.

Considering cause-specific mortality (table 3), in the fully adjusted model, underweight status was significantly associated with higher cancer mortality and lower mortality from digestive diseases, whereas obese status was significantly associated with lower cancer, digestive diseases and other-cause mortality. External-cause mortality was significantly higher in overweight and obese groups compared to those with healthy weight, and morbid obesity was strongly associated with respiratory disease mortality. None of the BMI groups was associated with circulatory disease mortality in either age-adjusted or fully adjusted models.

**Table 3:** Cox proportional model used for underlying cause-specific mortality by BMI category

BMI group	Circulatory HR (95% CI)	Cancer HR (95% CI)	Respiratory HR (95% CI)	Digestive HR (95% CI)	External causes HR (95% CI)	Any other causes HR (95% CI)
Model adjusted for age						
Healthy weight	Reference	Reference	Reference	Reference	Reference	Reference
Underweight	1.13 (0.71 to 1.81)	1.65 (0.98 to 2.78)	1.48 (1.01 to 2.38)	0.74 (0.34 to 1.60)	1.13 (0.66 to 1.93)	1.24 (0.80 to 1.95)
Overweight	1.15 (0.89 to 1.49)	0.87 (0.65 to 1.16)	1.28 (0.89 to 1.82)	0.83 (0.49 to 1.41)	0.96 (0.68 to 1.38)	0.81 (0.63 to 0.99)
Obese	0.98 (0.77 to 1.26)	0.74 (0.53 to 0.99)	0.73 (0.52 to 0.98)	0.71 (0.40 to 1.27)	1.01 (0.71 to 1.38)	0.72 (0.55 to 0.93)
Morbidly obese	1.32 (0.75 to 2.30)	1.14 (0.60 to 2.13)	1.56 (0.81 to 2.99)	0.79 (0.30 to 2.08)	0.78 (0.41 to 1.51)	1.07 (0.66 to 1.74)
Fully adjusted model						
Healthy weight	Reference	Reference	Reference	Reference	Reference	Reference
Underweight	1.03 (0.54 to 1.95)	1.93 (1.03 to 4.10)	0.94 (0.46 to 1.91)	0.15 (0.03 to 0.98)	1.52 (0.65 to 3.55)	1.54 (0.73 to 3.25)
Overweight	1.04 (0.74 to 1.46)	0.77 (0.51 to 1.14)	1.29 (0.79 to 2.12)	1.23 (0.48 to 3.13)	1.56 (1.02 to 2.77)	0.60 (0.42 to 0.86)
Obese	0.90 (0.65 to 1.26)	0.62 (0.40 to 0.97)	0.66 (0.41 to 1.04)	0.27 (0.09 to 0.85)	1.51 (1.01 to 2.74)	0.63 (0.43 to 0.90)
Morbidly obese	1.34 (0.63 to 2.84)	0.85 (0.40 to 1.80)	2.17 (1.02 to 5.22)	0.85 (0.09 to 7.94)	0.49 (0.17 to 1.40)	1.05 (0.56 to 1.97)

Fully adjusted model adjusted for age, gender, marital status, ethnicity, employment status, IMD, smoking status, HoNOS problems, received antipsychotic, antidepressant, antimanic agents, antihypertensives, lipid-lowering and antidiabetic medications, and comorbidities of diabetes, hypertension and hyperlipidaemia.

BMI, body mass index; CI, confidence interval; HoNOS, Health of the Nation Outcome Scale; HR, hazard ratio; IMD, Index of Multiple Deprivation.

When stratified by median follow-up time, for patients who had a longer follow-up time than the median length of the follow-up period in the study, the results showed that compared with the healthy weight group, mortality was significantly raised in the underweight group (HR: 2.01, 95% CI: 1.13 to 3.59) and lowered in the overweight group (HR: 0.84, 95% CI: 0.61 to 0.93). Patients who were followed up for less than the median length of time demonstrated that mortality was raised for the underweight group and lowered in the overweight group; however, they were not statistically significant (table 4).

**Table 4:** Cox proportional hazards model used for all-cause mortality by baseline recorded BMI stratified by median follow-up time (6.385 years)

BMI group	Follow-up time less than median HR (95% CI), P value	Follow-up time more than median* HR (95% CI), P value
Healthy weight	Reference	Reference
Underweight	1.19 (0.89 to 1.36), 0.341	2.01 (1.13 to 3.59), 0.018
Overweight	0.95 (0.80 to 1.02), 0.067	0.84 (0.61 to 0.93), 0.044
Obese	0.92 (0.77 to 1.10), 0.358	1.13 (0.83 to 1.53), 0.435
Morbidly obese	1.14 (0.83 to 1.56), 0.829	0.76 (0.39 to 1.15), 0.414

*Fully adjusted model adjusted for age, gender, marital status, ethnicity, employment status, IMD, smoking status, HoNOS problems, received antipsychotic, antidepressant, antimanic agents, antihypertensives, lipid-lowering and antidiabetic medications, and comorbidities of diabetes, hypertension and hyperlipidaemia.

BMI, body mass index; CI, confidence interval; HoNOS, Health of the Nation Outcome Scale; HR, hazard ratio; IMD, Index of Multiple Deprivation.

## Discussion

### Main findings

The WHO has identified six leading risk factors for mortality—overweight and obese status, high blood glucose, high cholesterol, high blood pressure, tobacco use and physical inactivity—which also increase the risk of chronic diseases.^19^ These risk factors may substantially account for the premature mortality recognised in patients with schizophrenia spectrum disorders; however, the relationship between body size and mortality among patients with schizophrenia spectrum disorders remains unclear, prior to this study.^19^ In this large cohort study, we observed non-linear and age-modified associations. Obesity categories were associated with higher mortality risk in patients aged 18–45 years; however, underweight status was the predominant risk factor in the sample overall, accounted for by its salience in patients over 65 years old. Although obesity is recognised as a risk factor for cardiovascular diseases,^20^ no associations were found with any BMI category and circulatory disease mortality in our sample; instead, associations, where present, were with respiratory disease mortality (for morbid obesity) and external-cause mortality (for overweight and obese status). The ‘obesity paradox’ describes the phenomenon that patients with an increasing BMI have a paradoxical decrease in mortality. Although obesity increases the risk of obesity-related diseases, overweight and obese status are associated with survival benefits. It is well recognised in epidemiological studies from other populations,^21^ and several studies in patients with chronic diseases have found J-shaped or U-shaped relationships between BMI and mortality.^22^

Associations between low BMI and mortality may be accounted for in part by the under measurement of potential confounding factors, such as smoking status,^23^ and in part by life-threatening disorders that give rise to weight loss.^24^ Our findings support this phenomenon in the fact that positive associations between underweight status and mortality were limited to the oldest age groups, positive association with respiratory disease mortality was reduced to the null in the adjusted model, and the strongest association was with cancer mortality and therefore, likely to reflect cancer-associated weight loss. Healthy survivor effects are another potential explanation for negative associations between obesity and mortality in older age groups.

Obesity and metabolic syndrome involving insulin resistance are common problems in patients with schizophrenia. The incidence of diabetes and cardiovascular diseases in patients with schizophrenia is significantly higher than expected, which underlies higher mortality and lower life expectancy.^25^ The associations we observed between obesity and mortality in the 18–45 years age group thus further underlie the importance of health improvement measures in these populations. Although cardiovascular outcomes are particularly concerning in this respect, we found no association with circulatory disease mortality, despite this being the most common cause of death. However, this may reflect the duration of follow-up applied in this cohort study; it is possible that consequences of obesity on respiratory disease mortality precede those on cardiovascular disease deaths (and that disorders underlying cardiovascular mortality may result in intervening weight loss). The effects of obesity on respiratory function lead to hypoxia, which results in an increased prevalence of respiratory diseases, such as obstructive sleep apnoea in obese people.^26^ However, the treatment of sleep apnoea in obese individuals can reduce the risk of cardiovascular disease.^27^

The rate of suicide, homicide and accidental death increases in these patient populations. For example, the lifetime risk of suicide in patients with schizophrenia has been estimated to be 5%–6%,^28^ and a UK cohort reported an absolute risk of suicide of 3.2% for patients 20 years after the initial diagnosis of a psychotic disorder.^29^ In our cohort, higher external-cause mortality was associated with overweight and obese status at baseline in the fully adjusted model. This requires further investigation as to the specific causes and underlying factors: for example, clarification as to whether this represents a direct effect of obesity or whether the obesity reflects a more severe underlying mental disorder.

Key strengths of this study include the study design, large sample size, relatively long-term follow-up and population representativeness. SLaM is one of the largest mental healthcare providers in Europe, serving a socially and ethnically diverse population within a geographical catchment area, thereby minimising selection bias. CRIS further provides ‘real-world’ information from complete routine clinical records, allowing a number of covariates to be ascertained that are not always available in cohorts derived from administrative data. Participants were followed up for up to 11 years and mortality ascertainment should be near complete from national certifications.

## Limitations

This study had some potential limitations. First, the SLaM catchment represents an inner urban to suburban area with limited generalisability to rural populations. However, there are no substantial differences in life expectancy estimates derived from serious mental illness groups in SLaM and those derived from other data resources.^30^ Second, it should be noted that the cohort consisted of people with schizophrenia spectrum disorders who had received specialist mental healthcare and would not necessarily generalise to those receiving primary care alone. Third, the cohort was further restricted to patients whose BMI had been measured and recorded, representing around half of those eligible by diagnosis. On examining differences between those included and not, unsurprisingly, the group of patients with a BMI measure were younger with potentially higher service involvement. Additionally, they had lower mortality than those without a BMI measure; therefore, generalisability needs to be further considered. Fourth, the patients who had BMI measured and recorded were more likely to receive holistic care and live longer. Fifth, because data were drawn from routine administrative and clinical records and not collected specifically for research purposes, issues of incompleteness, inaccuracy and inconsistency may have limited the scope to capture confounding variables. Finally, there are recognised limitations of BMI to measure obesity and the researches may need to consider other measures in further research, such as waist circumference and waist-to-hip ratio, although these are less routinely used in clinical practice.

## Implications

Schizophrenia spectrum disorders are accompanied by a range of potentially unhealthy lifestyles, such as poor diet, lack of physical exercise, smoking, illicit drug use, lack of self-care, and reduced access to disease prevention and screening programmes. These may be compounded by associations of antipsychotic agents with weight gain, metabolic problems and cardiovascular diseases. Consequently, premature mortality is an important concern.^5^ The impact of obesity on the life expectancy of people with schizophrenia spectrum disorders is clear from our findings in younger age groups, although it is also important to better understand underweight status as a risk factor later in life—in particular, whether this is simply a product of underlying comorbidity or whether there are factors relating to the mental disorder itself (eg, self-neglect) that might need further attention. There is an urgent need to adopt strategies to identify and prevent the causes of premature death. A deeper biological understanding of the relationship between these diseases and schizophrenia will help improve clinical practice.

## Data Availability

Data are available upon reasonable request.
