# Supplementary material for: Body mass index and mortality in patients with schizophrenia spectrum disorders: a cohort study in a South London catchment area
Source: Gen Psychiatr. 2022 Nov 4;35(5):e100819. doi: 10.1136/gpsych-2022-100819 (PMC9639123; doi:10.1136/gpsych-2022-100819)
Supplement: Supplementary data [file gpsych-2022-100819supp001.pdf]

Supplementary table: Comparison between included and non-included patients in the study cohort (number (%) or mean (SD))

Characteristics	Not included			Study cohort			Statistical comparisons		
	Alive (n= 9,187)	Deceased (n= 2,094)	Total (n= 11,281)	Alive (n= 10,334)	Deceased (n= 1,566)	Total (n=11,900)	test statistics, p-value Alive	deceased	Total
Age at the time of diagnosis (%)*							120.3,7, p<0.001	68.92,7, p<0.001	292.99,7, p<0.001
18- 24	1,142 (12.4)	33 (1.6)	1,175 (10.4)	1,809 (17.5)	57 (3.6)	1,866 (15.7)			
25- 34	2,408 (26.2)	119 (5.7)	2,527 (22.4)	2,776 (26.9)	134 (8.6)	2,910 (24.5)			
35- 44	2,362 (25.7)	231 (11)	2593 (23)	2,669 (25.8)	232 (14.8)	2,901 (24.4)			
45- 54	1,780 (19.4)	306 (14.6)	2,086 (18.5)	17,70 (17.1)	292 (18.6)	2,062 (17.3)			
55- 64	870 (9.5)	372 (17.8)	1,242 (11)	766 (7.4)	258 (16.5)	1,024 (8.6)			
65- 74	419 (4.6)	417 (19.9)	836 (7.4)	385 (3.7)	307 (19.6)	692 (5.8)			
75- 84	173 (1.9)	445 (21.3)	618 (5.5)	136 (1.3)	223 (14.2)	359 (3.0)			
85 and over	33 (0.4)	171 (8.2)	204 (1.8)	23 (0.2)	63 (4)	86 (0.7)			
Mean age at diagnosis (SD) [&]	41.1 (14.3)	62.7 (17.4)	45.1 (17.1)	38.9 (13.9)	57.2 (17.9)	41.3 (15.7)	10.9, 19519, p<0.001	9.3, 3658, p<0.001	17.6, 23179, p<0.001
Gender (%)*							15.6,1, p<0.001	0.01,1, p= 0.937	15.6,1, p<0.001
Female	3,928 (42.8)	956 (45.7)	4,884 (43.3)	4,131 (40.0)	717 (45.8)	4,848 (40.7)			
Male	5,258 (57.2)	1,138 (54.3)	6,396 (56.7)	6,203 (60.0)	849 (54.2)	7,052 (59.3)			
Missing	*1 (0.01)*	*0 (0.0)*	*1 (0.01)*	*0 (0.0)*	*0 (0.0)*	*0 (0.0)*			
Cohabiting status (%)*							1871.5,1, p<0.001	11.92,1, p<0.001	19.0,1, p<0.001
Cohabiting	1,142 (12.4)	292 (13.9)	1,434 (12.7)	1,134 (11)	159 (10.2)	1,293 (10.9)			
Non-cohabiting	8,045 (87.6)	1,802 (86.1)	9,847 (87.3)	9,200 (89)	1,407 (89.8)	10,607 (89.1)			

Living alone (%)	1,624 (17.7)	503 (24.0)	2,127 (18.9)	2,282 (22.1)	479 (30.6)	2,761 (23.2)			
Ethnicity (%)*							163.7,1, p<0.001	22.0,1, p<0.001	225.3,1, p<0.001
Non-white	4,379 (47.7)	701 (33.5)	5,080 (45.0)	6,159 (59.6)	661 (42.2)	6,820 (57.3)			
White	4,233 (46.1)	1,325 (63.3)	5,558 (49.3)	4,083 (39.5)	903 (57.7)	4,986 (41.9)			
Missing	*575 (6.3)*	*68 (3.2)*	*643 (5.7)*	*92 (0.9)*	*2 (0.1)*	*94 (0.8)*			
Employment status (%)*							402.8,4, p<0.001	77.9,4, p<0.001	486.4,4, p<0.001
Disabled	31 (0.3)	10 (0.5)	41 (0.4)	22 (0.2)	4 (0.3)	26 (0.2)			
Employed or student	354 (3.9)	12 (0.6)	366 (3.2)	503 (4.9)	34 (2.2)	537 (4.5)			
Other	2,869 (31.2)	727 (34.7)	3,596 (31.9)	2,803 (27.1)	471 (30.1)	3,274 (27.5)			
Retired	97 (1.1)	305 (14.6)	402 (3.6)	120 (1.2)	236 (15.1)	356 (3.0)			
Unemployed	1,425 (15.5)	434 (20.7)	1,859 (16.5)	3,160 (30.6)	552 (35.2)	3,712 (31.2)			
Missing	*4,411 (48.0)*	*606 (28.9)*	*5,017 (44.5)*	*3,726 (36.1)*	*269 (17.2)*	*3,995 (33.6)*			
Mean Index of multiple deprivation (SD) [&]	30.0 (11.2)	29.9 (10.8)	30.0 (11.1)	31.2 (9.6)	31.0 (10.0)	31.1 (9.6)	8.1, 19519, p<0.001	3.1, 3658, p=0.01	8.1, 23179, p<0.001
Smoking status (%)*							21.6,2, p<0.001	11.4,2, p=0.01	31.2,2, p<0.001
Current Smoker	3,762 (40.9)	874 (41.7)	4,636 (41.1)	6,897 (66.7)	956 (61)	7,853 (66.0)			
Ex-smoker	268 (2.9)	83 (4.0)	351 (3.1)	382 (3.7)	66 (4.2)	448 (3.8)			
Non-smoker	1,420 (15.5)	377 (18)	1,797 (15.9)	2,926 (28.3)	506 (32.3)	3,432 (28.8)			
Missing	*3,737 (40.7)*	*760 (36.3)*	*4,497 (39.9)*	*129 (1.2)*	*38 (2.4)*	*167 (1.4)*			
HoNOS problem (%)									
Agitation[$]	1,077 (11.7)	359 (17.1)	1,436 (12.7)	2,616 (25.3)	420 (26.8)	3,036 (25.5)	24.2, p<0.001	7.1, p<0.001	24.7, p<0.001
Self-injury[$]	427 (4.6)	60 (2.9)	487 (4.3)	686 (6.6)	93 (5.9)	779 (6.5)	6.0, p<0.001	4,6, p<0.001	7.5, p<0.001

Drinking$	841 (9.2)	171 (8.2)	1,012 (9.0)	1,958 (18.9)	259 (16.5)	2,217 (18.6)	19.5, p<0.001	2.6, p=0.01	21.2, p<0.001
Cognition$	869 (9.5)	474 (22.6)	1,343 (11.9)	1,928 (18.7)	405 (25.9)	2,333 (19.6)	18.3, p<0.001	2.3, p=0.03	16.1, p<0.001
Physical illness$	1,205 (13.1)	927 (44.3)	2,132 (18.9)	1,670 (16.2)	621 (39.7)	2,291 (19.3)	56.6, p<0.001	2.8, p=0.01	0.7, p= 0.49
Hallucinations$	2,714 (29.5)	802 (38.3)	3,516 (31.2)	5,431 (52.6)	843 (53.8)	6,274 (52.7)	32.5, p<0.001	9.3, p<0.001	33.2, p<0.001
Depression$	1,899 (20.7)	304 (14.5)	2,203 (19.5)	2,585 (25.0)	327 (20.9)	2,912 (24.5)	7.2, p<0.001	5.0, p<0.001	9.1, p<0.001
Relationships$	2,140 (23.3)	583 (27.8)	2,723 (24.1)	3,715 (35.9)	570 (36.4)	4,285 (36.0)	19.3, p<0.001	5.5, p<0.001	19.7, p<0.001
Daily living$	1,448 (15.8)	790 (37.7)	2,238 (19.8)	2,860 (27.7)	672 (42.9)	3,532 (29.7)	20.0,p<0.001	3.2, p<0.001	17.3, p<0.001
Living condition$	1,132 (12.3)	322 (15.4)	1,454 (12.9)	2,293 (22.2)	347 (22.2)	2,640 (22.2)	40.8,p<0.001	5.3 , 0<0.001	18.6, p<0.001
Occupation$	1,670 (18.2)	570 (27.2)	2,240 (19.9)	3,199 (31)	512 (32.7)	3,711 (31.2)	20.6, p<0.001	3.6 , p<0.001	19.7, p<0.001
Psychiatric medication (%)									
Antipsychotic$	6,221 (67.7)	1,495 (71.4)	7,716 (68.4)	9,473 (91.7)	1,380 (88.1)	10,853 (91.2)	42.1, p<0.001	12.2,p<0.001	43.5, p<0.001
Antidepressant$	2,960 (32.2)	634 (30.3)	3,594 (31.9)	4,155 (40.2)	628 (40.1)	4,783 (40.2)	11.6, p<0.001	6.2, p<0.001	13.2, p<0.001
Anxiolytic/hypnotic$	2,779 (30.2)	584 (27.9)	3,363 (29.8)	6,341 (61.4)	848 (54.2)	7,189 (60.4)	43.5, p<0.001	16.2, p<0.001	46.8, p<0.001
Mood stabiliser$	752 (8.2)	193 (9.2)	945 (8.4)	1,910 (18.5)	333 (21.3)	2,243 (18.8)	20.9, p<0.001	10.3, p<0.001	23.1, p<0.001
Medication for CVD (%)									
Antihypertensive$	81 (0.9)	48 (2.3)	129 (1.1)	137 (1.3)	65 (4.2)	202 (1.7)	2.94, p=0.01	3.2 ,p=0.01	3.6, p=0.01
Lipid lowering$	475 (5.2)	318 (15.2)	793 (7.0)	836 (8.1)	343 (21.9)	1,179 (9.9)	80.3, p<0.001	5.2, p<0.001	7.9, p<0.001
Antidiabetic$	149 (1.6)	135 (6.4)	284 (2.5)	352 (3.4)	166 (10.6)	518 (4.4)	7.8, p<0.001	4.5, p<0.001	7.6, p<0.001
Hospitalized diseases (%)									
Diabetes$	326 (3.5)	318 (15.2)	644 (5.7)	295 (2.9)	183 (11.7)	478 (4.0)	2.75, p=0.01	3.0, p=0.01	6.0, p<0.001
Hypertension$	494 (5.4)	498 (23.8)	992 (8.8)	350 (3.4)	268 (17.1)	618 (5.2)	6.8, p<0.001	4.9, p<0.001	10.8, p<0.001
Hyperlipidaemia$	212 (2.3)	182 (8.7)	394 (3.5)	135 (1.3)	111 (7.1)	246 (2.1)	5.3, p<0.001	1.8, p=0.08	6.6, p<0.001
BMI category (%)									
Healthy weight (18.5 to 24.9)				4,079 (39.5)	580 (37)	4,659 (39.2)			
Underweight (10 to 18.4)				457 (4.4)	98 (6.3)	555 (4.7)			

Overweight (25.0 to 29.9)				2,980 (28.8)	423 (27)	3,403 (28.6)			
Obese (30 to 39.9)				2,388 (23.1)	394 (25.2)	2,782 (23.4)			
Morbidly obese (40 to 49.9)				430 (4.2)	71 (4.5)	501 (4.2)			
Mean BMI (SD)				26.9 (6.2)	27.1 (6.6)	26.9 (6.3)			
Mean years of follow-up (SD) [&]	8.2 (3.6)	3.9 (3.3)	7.4 (3.5)	8.9 (3.6)	6.2 (3.4)	8.6 (3.5)	13.6, 19519, p<0.001	20.6, 3658, p<0.001	26.1,23179, p<0.001
Cause of death (%)									
Circulatory					291 (18.6)				
Cancer					200 (12.8)				
Respiratory system					166 (10.6)				
Digestive system					71 (4.5)				
External causes					158 (10.1)				
Other					680 (43.4)				

*statistical comparisons were made using chi squared test. Chi squared value, degree of freedom and p-values are displayed.

[&] statistical comparison were made using student t-test. T-test value, degree of freedom and p-values are displayed.

[$] statistical comparison were made using difference in proportions test. Z value and p-values are displayed.
